# Phylogenetic insights into the early spread of the SARS-CoV-2 Alpha variant across Europe

**DOI:** 10.1093/ve/veaf030

**Published:** 2025-06-25

**Authors:** Abbey Evans, Damien C Tully

**Affiliations:** Department of Infectious Disease Epidemiology, London School of Hygiene and Tropical Medicine, London WC1E 7HT, United Kingdom; Department of Infectious Disease Epidemiology, London School of Hygiene and Tropical Medicine, London WC1E 7HT, United Kingdom; Centre for the Mathematical Modelling of Infectious Diseases, London School of Hygiene and Tropical Medicine, London WC1E 7HT, United Kingdom

**Keywords:** SARS-CoV-2, Alpha variant, phylogenetics, genomic surveillance, Europe

## Abstract

The evolution of Severe Acute Respiratory Syndrome Coronavirus 2 (SARS-CoV-2) has profoundly influenced the trajectory of the COVID-19 pandemic. In late 2020, the Alpha variant (Pango lineage B.1.1.7) emerged in South East England, characterized by enhanced transmissibility, increased mortality, and rapid geographic expansion. Here, we studied the evolutionary history and migration dynamics of Alpha across Europe using genomic data sourced from 38 European countries. Our findings reveal that Alpha was introduced to over 25 European countries within 90 days of its emergence, with the UK accounting for 61% of early exportation events. However, as the epidemic progressed, several mainland European countries, including France, Denmark, and the Czech Republic, became significant hubs of onward transmission. Social mixing during the December holiday period and air travel facilitated the variant’s rapid dissemination, as corroborated by air passenger flight volumes and viral introductions. Notably, genomic surveillance intensified after Alpha was designated a variant of concern, reducing the detection lag in countries with later introductions. Our study highlights the critical interplay between international mobility, surveillance efforts, and regional connectivity in shaping the epidemiology of SARS-CoV-2 variants and underscores the need for coordinated genomic surveillance and timely interventions to mitigate the spread of emerging pathogens.

## Introduction

Since the emergence of Severe Acute Respiratory Syndrome Coronavirus 2 (SARS-CoV-2), the aetiological agent of coronavirus disease 2019 (COVID-19), concerns have arisen over emerging variants with increased transmissibility. In December 2020, an investigation by Public Health England identified an unusual genomic cluster in South East England, coinciding with a rapid increase in COVID-19 cases (Public Health England 2020). Subsequent evidence demonstrated that this cluster was rapidly expanding across England between November 2020 and January 2021 during a national lockdown. Under the Pango lineage classification system, this cluster was designated B.1.1.7 (Rambaut et al. 2020) and later classified as a variant of concern (VOC) under the World Health Organisation variant nomenclature (Konings et al. 2021). Intensive research has since revealed that the rapid growth of this lineage was driven by human mobility and super-seeding events from its source location (Kraemer et al. 2021). Additionally, its increased transmissibility (Davies et al. 2021a) contributed to greater mortality among community cases (Davies et al. 2021b) and an increased risk of hospitalization compared with other lineages (Bager et al. 2021).

The B.1.1.7 lineage was particularly unusual due to its high divergence from its nearest common ancestor, having accumulated a constellation of 17 mutations—14 nonsynonymous mutations and 3 deletions—compared to contemporaneous lineages (Rambaut et al. 2020). Eight of these notable mutations were found in the spike protein, including the N501Y mutation. This mutation enhances binding to human ACE2 receptors by up to 3.5-fold and has been linked to increased infectivity and transmissibility (Starr et al. 2020, Yuan et al. 2021). Due to convergent evolution, the N501Y mutation has also been identified in multiple distinct variants, including Gamma (P.1) (Faria et al. 2021), Beta (B.1.351) (Tegally et al. 2021) and the highly transmissible Omicron (B.1.1.529) variant along with its accumulating sublineages (Viana et al. 2022). Although B.1.1.7 was first sampled in Kent, South East England, in September 2020, its precise origin remains an evolutionary puzzle. Several plausible explanations exist. One possibility is cryptic transmission in regions with limited genomic surveillance. Another hypothesis suggests reverse zoonosis, where SARS-CoV-2 jumps from humans to non-human animals, such as white-tailed deer (Pickering et al. 2022) or mink (Munnink et al. 2021), evolves within these hosts, and subsequently re-merges in human populations. However, a more widely accepted theory is that B.1.1.7 evolved within a chronically infected individual, such as an immunocompromised individual (Hill et al. 2022). Such an environment would allow the virus to undergo prolonged evolutionary adaptation, potentially giving rise to new variants.

Previous studies have investigated the early spatiotemporal dynamics of the Alpha variant in various regions, including the USA (Alpert et al. 2021, Washington et al. 2021), the UK (Kraemer et al. 2021) and Denmark (Michaelsen et al. 2022). A more recent study has quantified the silent spread of Alpha out of the UK using a Bayesian framework (Faucher et al. 2024). However, the extent to which European countries contributed to the seeding and onward transmission of Alpha within Europe remains unclear, despite 45 countries reporting cases of B.1.1.7 as early as January 2021 (O’Toole et al. 2021).

In this study, leveraging the wealth of genomic data available for Alpha, we applied phylogenetic and phylogeographic methods to examine its dispersal patterns across Europe. Our aim was to determine the source-sink dynamics of individual countries and better understand the role of cross-border transmission in the spread of Alpha.

## Methods

### Genomic data and sampling

All available full-length SARS-CoV-2 sequences (*n* = 2 004  490) and corresponding metadata were downloaded from the GISAID database (https://www.gisaid.org/) as of 22 June 2021. The NextStrain Augur based pipeline (Hadfield et al. 2018) was used to perform quality control and phylogenetic analysis. In brief, sequences were filtered to collection dates between 20 September 2020 and 31 January 2021 (*n* = 501 554) to capture the emergence of B.1.1.7 within England as well as the early introductions and establishment of community transmission in each European country. Sequences were filtered to include only those designated as Pango lineage B.1.1.7 (*n* = 87 847) according to the Pango dynamic lineage nomenclature scheme (O’Toole et al. 2021) and designated as from the European region. Incomplete genomes, those from non-human hosts, duplicate sequences, and those with incomplete information on the date or location of collection were excluded. In order to minimize any selection bias, we produced 10 subsampled datasets generated from probabilistic sampling using unique random number seeds for each dataset. Each dataset was ∼20 000 sequences in size. Sequences were aligned using MAFFT v.7.470 (Katoh and Standley 2014), and the resulting alignment was trimmed at the 5ʹ and 3ʹ regions. Three known homoplasmic positions (13 402, 24 389, and 24 390) were masked in the alignment. Datasets were also compiled for ORF1a, the spike surface glycoprotein and the nucleocapsid phosphoprotein.

### Phylogenetic reconstruction

For each of the 10 replicates, a maximum likelihood tree was created using IQ-Tree version 2.2.2.2 (Minh et al. 2020) using the best fit model of nucleotide substitution according to Bayesian information criterion. Maximum likelihood trees were transformed into time-scaled phylogenies using TreeTime (Sagulenko et al. 2018) with a clock rate set to 0.0008 substitutions per site per year and a standard deviation of 0.0004. To ensure that polytomies were resolved adequately, TreeTime was run with 10 iterations for each dataset. Temporal outliers were flagged and removed, and TreeTime was re-run until a time-scaled phylogeny free of outliers was obtained. A migration model was fitted to each of the time-calibrated tree topologies in TreeTime, mapping the country location of sampled sequences to the external tips of the trees. The migration model of TreeTime infers the most likely location for internal nodes in the trees, and the number of state changes can be counted by iterating over each phylogeny from the root to the external tips. State changes are then counted when an internal node transitions from one country to a different country in the resulting child node or tip(s). The timing of such state transition events is then recorded, which serves as the estimated import or export event. Any state transitions that occurred prior to the earliest known time to the most recent common ancestor (tMRCA) were removed to avoid any transitions with low confidence. The spike, nucleocapsid, and ORF1a were also subjected to the same phylogenetic analyses and compared to full-length SARS-CoV-2 genomes to assess any differences in migration events.

### Air travel data

Publicly available air passenger traffic data was procured from the UK’s Civil Aviation Authority from September to January 2021. As Alpha emerged in the South East of England, only data from London airports (Gatwick, Heathrow, London City, Luton, Southend, and Stansted) were considered. Data from each airport were aggregated to give a pooled total number of outbound passengers from the Greater London region to each European country per month. Chartered passenger flights were included in the pooled totals, and cancelled bookings were excluded. Correlations with air travel passenger volumes were calculated using the Spearman rank correlation methods with the level of significance reported.

## Results

### Severe Acute Respiratory Syndrome Coronavirus 2 Alpha genomic data

**Figure 1.**
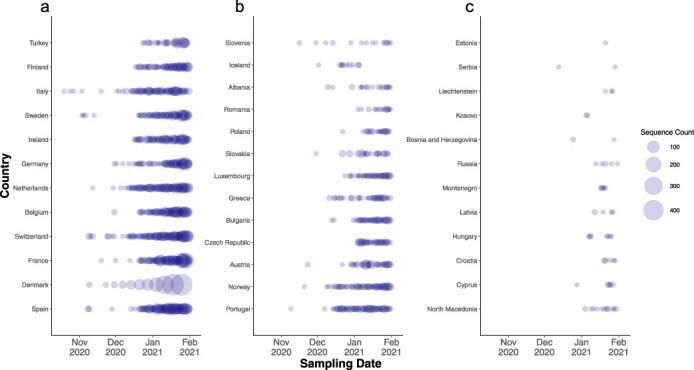
**Temporal distribution of Alpha sequences across European countries**. The bubble plot represents the number of sequences sampled per country over time. Each bubble corresponds to the count of sequences identified on a given date, with bubble size proportional to the sequence count. *X*-axis denotes the sampling date from September 2020 to February 2021 while the *y*-axis lists countries ranked by total sequence count. The plot is divided into there panels with countries grouped based on the volume of sequences generated. (a) Countries with the highest sequence counts, (b) countries with intermediate sequence counts, and (c) countries with the lowest sequence counts.

As of 22 June 2001, 92 826 SARS-CoV-2 genomes ascribed as Pango lineage B.1.1.7 or Alpha had been submitted to the GISAID database from 38 European countries. After filtering and quality control 87 487 SARS-CoV-2 genomes passed and met the minimum metadata requirements and were sampled between September 2020 and 31 January 2021 (Fig. 1). Overall, 85% of the genomes were sampled in the UK with the earliest sampled sequence corresponding to the 20th of September 2020. Outside of the UK, Spain, Denmark, and France had the highest number of samples with 1651, 1626, and 1610 sequenced genomes, respectively (Fig. 1a). The earliest sequenced genome outside of the UK came from Italy on the 19th of October 2020 followed by Sweden, Spain, Denmark, Switzerland, Portugal, and the Netherlands who all had sequenced Alpha positive cases within the first 2 weeks of November (Fig. 1a). Slovenia, France, Norway, Austria, Slovakia, Belgium, and Germany had their earliest sequenced genomes in the last 2 weeks of November. Most European countries had sequenced Alpha samples in November or December 2020 (Fig. 1a and b) with the volume of sequences generated varying between those sequencing 500 or more cases (Fig. 1a) or those countries with 300 genomes or less (Fig. 1b). Estonia, Serbia, Liechtenstein, Kosovo, Bosnia and Herzegovina, Russia, Montenegro, Lativa, Hungary, Croatia, Cyprus, and North Macedonia all had <20 sequences generated, at the time of analysis, which were generally sampled in January of 2021 (Fig. 1c). Together, over 90% of all European genomes were deposited in January of 2021 suggesting intensified surveillance and sequencing efforts after the announcement of B.1.1.7 as a VOC.

To quantify the dissemination pattern of Alpha across Europe and to minimize the overrepresentation of sequences from countries with the highest sequencing efforts, such as the UK, we randomly subsampled sequences to produce datasets of ∼20 000 sequences for each replicate (Table 1). This subsampling scheme was performed 10 times using 10 unique random number seeds to produce 10 randomly sampled genomic datasets for Alpha ensuring that genomes from all 38 countries (Albania, Austria, Belgium, Bosnia and Herzegovina, Bulgaria, Croatia, Cyprus, Czech Republic, Denmark, Estonia, Finland, France, Germany, Greece, Hungary, Iceland, Ireland, Italy, Kosovo, Latvia, Liechtenstein, Luxembourg, Montenegro, Netherlands, North Macedonia, Norway, Poland, Portugal, Romania, Russia, Serbia, Slovakia, Slovenia, Spain, Sweden, Switzerland, Turkey, and the UK) were sampled.

**Table 1.** Overview of genomic subsampling datasets.

Replicate	Seed number	Initial no. of sequences	Final no. of sequences
1	5007	20 012	19 968
2	687	20 012	19 875
3	2581	20 010	19 919
4	4294	20 011	19 967
5	7380	20 010	19 963
6	8111	20 010	19 898
7	2973	20 011	19 967
8	91	20 011	19 978
9	3226	20 010	19 962
10	5	20 012	19 978

## Quantifying the viral exchanges from the UK and within Europe

**Figure 2.**
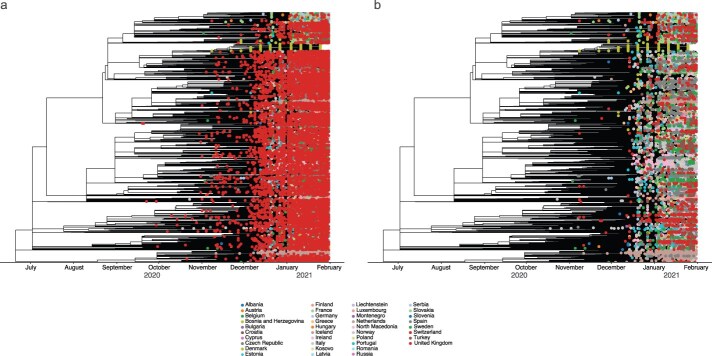
**Phylogenetic reconstruction of Alpha SARS-CoV-2 sequences on the continent of Europe**. (a) Time-resolved maximum likelihood tree containing 19 978 full-length genome sequences and (b) time-resolved maximum likelihood tree containing full-length genome sequences minus the UK to highlight country-level clusters. Genomes of specific countries are denoted in various colours as indicated in the legend.

**Figure 3.**
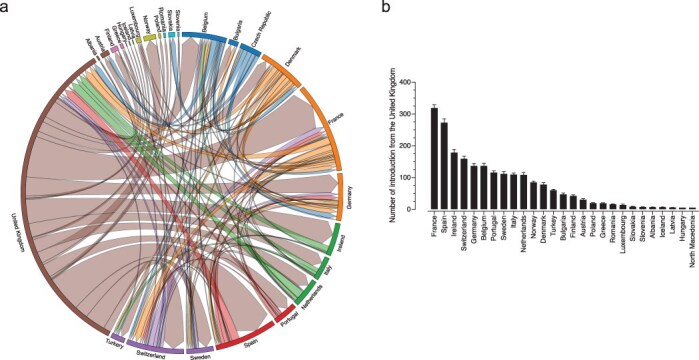
**Inferred viral dissemination patterns of SARS-CoV-2 Alpha between the UK and European countries**.(a) Circular migration flow plot based on the phylogenetic inferred number of migration flows between the UK and other European countries. Dissemination patterns are obtained from inferred ancestral-state reconstructions. The size of the plot reflects the total number of transitions for that country. Migration flow out of a particular country starts close to the outer ring and ends with an arrowhead more distant from the destination location. (b) The number of transitions from the UK into European countries. Error bars are determined from 10 replicates of genomic subsampling.

Discrete state maximum likelihood reconstruction from 10 subsampling subsets inferred at least 3444 (95% CI: 3399–3487) location transition events within Europe from September 2020 to January 2021. To gain insights into the early stage of Alpha spread in Europe, we traced the geographical sources of viral introductions to reveal dispersal patterns between European countries and found that successful transmission lineages arrived early and spread widely (Fig. 2). We found that a median of 2102 (95% CI: 2046–2128) or 61% of migration events were attributed with originating from the UK (Fig. 3a). While the UK was a predominant net exporter, several viral importer events into the UK were detected. The top country sources of importation were Spain, the Netherlands, Ireland, and France. By calculating the count of introduction and exportation events from the UK we observed that France had the highest number of importation events [mean of 319, ±9.8 (standard deviation)], followed by Spain (mean of 272, ±11.9), Ireland (mean of 178, ±9.6), Switzerland (mean of 158.9, ±8), Germany (mean of 136, ±7.6), Belgium (mean of 136, ±8.6), Portugal (mean of 115, ±5.7), Sweden (mean of 111, ±7.3), Italy (mean of 109, ±4.8), and the Netherlands (mean of 108, ±7.9) (Fig. 3b). The remaining 17 countries all had an average of <100 introductions ranging from 5 (North Macedonia and Hungary) to 84 for Norway.

**Figure 4.**
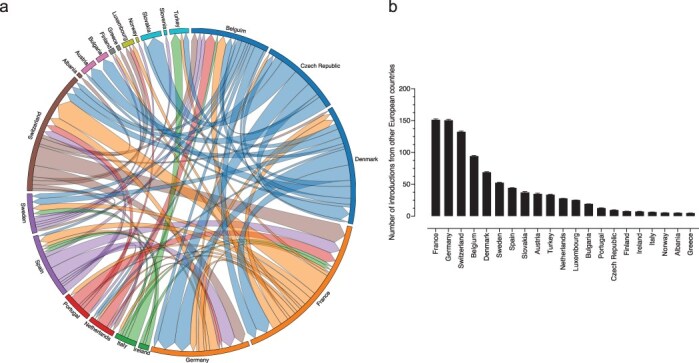
**Inferred viral dissemination patterns of SARS-CoV-2 Alpha within European countries**.(a) Circular migration flow plot based on the phylogenetic inferred number of migration flows between countries except the UK. Dissemination patterns are obtained from inferred ancestral-state reconstructions. The size of the plot reflects the total number of transitions for that country. Migration flow out of a particular country starts close to the outer ring and ends with an arrowhead more distant from the destination location. (b) The number of transitions from European countries. Error bars are determined from 10 replicates of genomic subsampling.

To get a more comprehensive view of SARS-CoV-2 Alpha exchanges between other European countries, we focused on European countries excluding the UK (Fig. 4a; Supplementary Figure 1). France was estimated to have contributed 16% of the total exportation events across Europe, followed by the Czech Republic (13.76%), Denmark (12.96%), Switzerland (12.27%), and Spain (9.48%). France appeared to act as a major hub for the spread of Alpha within Europe and was the primary source of exchange for countries such as Belgium, Switzerland, Spain, Sweden, and the Netherlands. It was also the second highest source of importation for Denmark. Interestingly, France and Switzerland had the highest reciprocal number of exportations towards each other. The Czech Republic demonstrated notable export activity and was a main exporter to parts of Europe, having the highest ratio of exportation to importation events, which resulted in several migration events to bordering countries such as Germany, Slovakia, and Austria. The Czech Republic also participated in exporting the virus to Denmark and Switzerland, while Germany was mostly seeded from Switzerland, Denmark, Spain, and France. While viral migration patterns were influenced by geographical proximity, it was not always the case that neighbouring countries were the primary contributors in terms of viral exchange. Spain and Portugal had minimal reciprocal exchanges, while in Northern Europe, there was limited evidence of introductions through neighbouring countries with Denmark and Sweden appearing to seed each other at the same rate and to a smaller degree Norway and Finland. For other European countries, there were fewer exchange events. For example, in Slovakia and Austria the main source of introduction events stemmed almost exclusively from Czech Republic. The main source of exchanges for the Benelux countries was often from its neighbouring states. The Netherlands appeared to contribute more to virus exportation towards Belgium, which was only responsible for a relatively small number of seeding events to the Netherlands and Luxembourg.

Examination of exportation events outside the UK revealed that France has the highest number of importation events (mean of 151, ±1.3), from other European countries closely followed by Germany (mean of 151, ±1.4), Switzerland (mean of 133, ±1.1), and Belgium (mean of 94.2, ±0.8) (Fig. 4b). Notably, Ireland exported very few events towards Europe and almost exclusively only had imported events from the UK (Figs 3 and 4). Analyses of specific genomic regions revealed a consistent pattern of migrations events (Supplementary Figure 2). Although more migration events were recorded for some proteins, such as Spike, these unique events were relatively low in abundance. Thus, the major transmission patterns inferred from the full genome analyses hold true for specific genes.

### Determining the number, timing, and origin of Alpha importations into Europe

**Figure 5.**
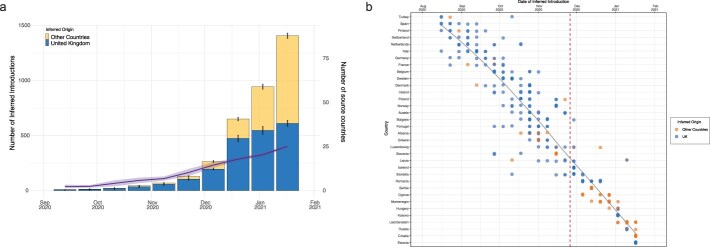
**Phylogenetic inference of Alpha dissemination events**.(a) The number of inferred introductions from the UK or another European country over time. The purple line indicates the number of countries inferred to be acting as an onward source of transmission (scale in the second *y*-axis). Results are determined from 10 replicates of genomic subsampling as detailed in the methods. (b) Date of first inferred introduction per country coloured according to the presumed location of origin i.e. presumed origin from the UK indicated by blue or from another European country as orange. The lines represent countries, which are ordered based on the median date of the first inferred introductions with the country experiencing the earliest median introduction at the top and the latest at the bottom. Vertical dashed red line represents 100 days after the estimated tMRCA of the Alpha variant. Solid grey line represents the smoothed conditional mean line using the LOESS method.

Over time, the inferred sources of SARS-CoV-2 Alpha variant introductions shifted significantly. By the end of December 2020, ∼73% of introductions were estimated to originate from the UK. However, by mid-January 2021, there was a substantial change, with 57% of introductions inferred to come from outside the UK (Fig. 5a). This shift indicates that other European countries increasingly became responsible for the onwards transmission of the Alpha variant. Additionally, the number of countries identified as sources of transmission rose from a minimum of 10 in November 2020 to at least 25 by the end of January 2021 (as illustrated by the purple line in Fig. 5a).

An analysis of the timing of the first inferred introductions from 34 countries revealed that by December 2020, at least 25 European countries had at least one inferred introduction of the variant within the first 100 days after the estimated tMRCA of Alpha (Fig. 5b). Following these 100 days, most initial introduction to other countries (e.g. Cyprus) were inferred have originated from locations other than the UK. Turkey, Spain, Switzerland, and Finland all had multiple replicates, which indicated introductions as early as late August to mid-September. A cluster of introductions was observed in Central and Northern Europe from mid-September to late October, involving countries like the Netherlands, Italy, Germany, France, Belgium, Sweden, Denmark, Ireland, Poland, and Norway. In November, another wave of introductions was found to have seeded a wider geographic range including Austria, Bulgaria, Portugal, Albania, Greece, Slovenia, Luxembourg, Latvia, Slovakia, and Iceland. Romania, Cyprus, Montenegro, Kosovo, Hungary, Liechtenstein, Russia, Croatia, and Estonia all experienced later introductions from early December 2020 to mid-January 2021. The period around October to November was when Alpha seeded most European countries for the first time and before any designation of its status as a VOCs and before non-pharmaceutical interventions were extended. Additionally, we calculated the detection lag (number of days between the inferred tMRCA and the first sequenced sample) with a median of 25.75 days (IQR: 4–57.75 days) and noted that this varied depending on country (Supplementary Figure 1). Countries that were seeded early had a longer lag time (e.g. Turkey, Finland, and Spain) before the Alpha variant expanded. In contrast, countries which experienced a later inferred introduction, such as those in early January 2021 (e.g. Russia, Croatia, and Estonia), had a decreased lag time, probably due to heightened genomic surveillance efforts attributed to the designation of this lineage as a VOC.

### Impact of air travel and the importation of Alpha from the UK to Europe

**Figure 6.**
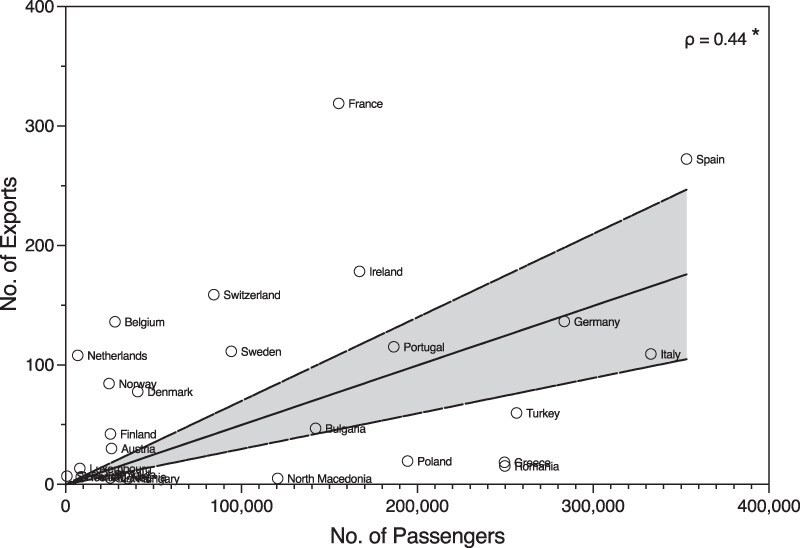
**Contribution of air travel between European countries and inferred exportation numbers**. Scatter plot denoting the number the volumes of air travel passengers and inferred numbers of Alpha exportation for each country. Spearman rank correlation values are shown, with the level of significance indicated.

To examine if air travel has influenced the velocity of Alpha dispersal, we investigated air travel passenger volumes between September 2020 and January 2021 and compared them with the number of introductions found in each country using genomic data. We find that the number of UK exportations is positively correlated with countries total air travel passenger volume (Spearman correlation *ρ* = 0.44, *P* < .02, Fig. 6). This is consistent with our phylogenetic analysis that the dispersal of Alpha into European countries was initially largely due to seeding from UK sources followed by more sustained transmission between European countries.

## Discussion

Using a set of over 20 000 Alpha genomic sequences sampled across Europe, we were able to disentangle the early importation and spread of SARS-CoV-2 in Europe. We revealed that early transmission predominantly stemmed from the UK, accounting for 61% of migration events, with France, Spain, and Denmark also playing significant roles. Importation events were frequent from Spain, the Netherlands, and France to the UK, while other European countries, such as France and the Czech Republic, acted as hubs for intra-European transmission. Over time, the UK’s role as the primary source waned, with 57% of Alpha introductions by mid-January 2021 attributed to non-UK origins. Within 100 days of its emergence, the Alpha variant, was introduced into 74% of European countries arising from the abolishment and loos of lockdown measures around the Christmas holidays. Increased genomic surveillance following Alpha’s designation as a VOC improved detection but revealed significant lags in those countries that were seeded early. Overall, our comprehensive phylogenetic analyses unveiled a network of intricate viral exchanges between neighbouring countries, while also uncovering non-proximal exchanges which may have been driven by factors beyond air travel volume, which played a significant role in contributing to the early dissemination and expansion of SARS-Cov-2 variants of concern (Tegally et al. 2023). These findings describe how most migration events between countries stem from asymmetrical transmission patterns, which was a noted observation with the emergence of Alpha within the UK (Kraemer et al. 2021).

In this study, we make phylogenetic inferences that provide detailed information on the timing and early spread of the SARS-CoV-2 Alpha variant across mainland Europe. We conclude that Alpha was introduced in more than 25 countries within 90 days of its emergence and before the international community was put on alert on 18 December 2020. The Alpha variant has been extensively studied from a phylodynamic and modelling perspective, where it was shown that Alpha had a substantial transmission advantage over other circulating lineages (Volz et al. 2021, Davies et al. 2021a). The origins of this lineage have also been carefully assessed, where it thought that the emergence of Alpha was from an individual chronically infected with SARS-CoV-2 over the course of months (Hill et al. 2022). Within Europe, the importance of regional historical and economic ties between countries such as West Poland and East Germany revealed a shared high frequency of transmission networks (Serwin et al. 2022). However, our analyses did not reveal any exchanges between these countries as our study was focused on the early introduction of Alpha while the latter study focused on a longer timeframe to May 2021. An Alpha sub-lineage characterized by additional mutations of nucleoprotein G204P and open-reading-frame-8 K68stop has been identified as spreading through several European countries such as Czech Republic, Austria, and Slovakia (Stadtmüller et al. 2022). Within the Czech Republic this sub-lineage quickly displaced other lineages escaping local control measures and was shown to be a high-exporter to other European countries (Stadtmüller et al. 2022). This finding agrees with our results, which demonstrate that the Czech Republic acted as a major hub in spreading Alpha throughout many European countries. In Denmark, a major investment in testing and sequencing led to extensive and unique phylogenetic insights on the timing and evolution of SARS-CoV-2 variants and the role that different age groups played in transmission (Khurana et al. 2024). Denmark was also the second country to report the Alpha variant (2 December 2020) and an in-depth analysis of over 60 000 SARS-CoV-2 genomes representing 34% of all positive cases from November 2020 to February 2021 in Denmark corroborated early estimates of increased transmissibility of Alpha and revealed an early introduction of the variant with substantial local within-region transmission before onwards spread to all other Danish regions within weeks (Michaelsen et al. 2022). Our results complement this prior work by confirming an early introductory time of the virus into Denmark with a short lag time in detection. Similarly, in the Netherlands the estimated timing of introduction of Alpha was estimated to be relatively early (mid–late September) where most introductions from within Europe were from neighbouring countries such as France and Belgium which is line with a prior study examining the importation and spread of SARS-CoV-2 variants within the Netherlands (Han et al. 2022). An investigation into the dissemination of Alpha into northwest Spain revealed that initial introductions were predominantly from other Spanish regions and France (Gallego-García et al. 2024). Notably, France was also the largest exporter of Alpha from our analyses on country-wide estimates of Spain.

Our study is affected by several limitations. Firstly, inherent biases due to heterogenous genomic surveillance efforts exist and has been extensively discussed (Brito et al. 2022). To address these issues we carefully downsampled countries which contributed disproportionately to the dataset (e.g. UK) (see Fig. 1). We repeated our phylogenetic inferences across 10 replicates of ∼20 000 genomes to mitigate any source of bias. Secondly, given the scale of our datasets and to ensure computational tractability, it was not possible to perform a full Bayesian phylogenetic and phylogeographic reconstruction. Thirdly, the mutational rate of SARS-CoV-2 coupled with unequal sampling rates can further complicate phylogeographic inferences of introduction sources and illustrate the need for combining different data types such as travel history (Villabona-Arenas et al. 2020), which can now be integrated into Bayesian phylogeographic inferences (Lemey et al. 2020). Fourthly, we only examined genomic sequence data while we recognize that other diverse sources of data if available should be considered for a finer interpretation on the dissemination of variants of concern (Faucher et al. 2024). A more comprehensive analysis that examines specific geographic factors, national epidemic control policies may elucidate a finer resolution on more precise routes of dissemination, and we recognize that our inferred networks of viral dispersal solely based on phylogenetic estimates are undoubtedly influenced by the highly heterogeneous context of local epidemics which are influenced by factors such as epidemic sizes, mobility patterns and levels of population immunity.

The size of SARS-CoV-2 genes varies in range meaning that key phylogenetic information from smaller genes may be obscured when examining full-length SARS-CoV-2 genomic sequences (Li et al. 2020). To examine whether there is an effect of gene length disparities on phylogenetic reconstruction and dissemination patterns, we performed additional phylogenetic analyses on three major genomic regions: the spike surface glycoprotein, nucleocapsid, and ORF1a and compared the migration events inferred from individual genomic segments to our full-genome analysis. Our analyses of specific segments of the SARS-CoV-2 genomes reveal a strong overlap of migration patterns between countries (76%–84%; Supplementary Figure 2) although some unique events were observed, these were found in relatively low abundance. Given the non-uniform rates of evolution of SARS-CoV-2 proteins, future work is needed to assess the reliability of utilizing full-length genomes in phylogenetic-based inferences of transmission.

In conclusion, this study measures the contribution of individual countries to the early spread of the Alpha variant. Although the Alpha variant has been extensively studied from an epidemiological and genomic landscape, our analyses shed further insights into the spatial and temporal patterns of viral spread from a broader European-wide genomic perspective. This information is crucial from a public health and policy perspective to understand how from a regional dimension geographic proximity and social connectivity play an integrated role into the dynamics of SARS-CoV-2 transmission. Moreover, investigating the early spread of SARS-CoV-2 variants is important to assess the effectiveness of any early interventions and the design of future COVID-19 control measures. This study also illustrates that the international response of lockdown measures, heighted screening, and travel bans arrived too late for countries to prevent containment and would only delay the time to dominance of the new variant by a matter of days, as illustrated by phylogenetic and modelling analyses (Reichmuth et al. 2023).

## Supplementary Material

veaf030_Supp

## Data Availability

All genome sequences and associated metadata in this dataset are published in GISAID’s EpiCoV database. To view the contributors of each individual sequence with details such as accession number, Virus name, Collection date, Originating Lab and Submitting Lab, and the list of Authors, visit 10.55876/gis8.241212wy.
