# Supplementary material for: Phylogenetic insights into the early spread of the SARS-CoV-2 Alpha variant across Europe
Source: Virus Evol. 2025 Jun 25;11(1):veaf030. doi: 10.1093/ve/veaf030 (PMC12223989; doi:10.1093/ve/veaf030)
Supplement: veaf030_Supp [file veaf030_supp.zip › suppl_data/Supplementary Figure 1-2.docx]

**Supplementary Figure 1. Detection lag between inferred dates of introduction per country as determined by phylogenetic analysis and date of first sequenced sample.** The earliest sequenced dates according to GISAID are depicted as a black circle and the inferred date of introductions estimated by phylogenetic analyses are shown either in grey for the mean date between replicates or in red for the median date between replicates.

**Supplementary Figure 2. Comparison of migration events between countries across datasets. (A)** The percentage of total migration events in which each country is recorded as the source (origin) in four datasets: Nucleocapsid (green), Spike (Blue), ORF1a (Purple) and Full Genome (Red). **(B)** Corresponding percentages for countries acting as the destination for migration events in the same datasets. Only countries contributing 1% or greater migration events are included.
