# Supplementary figures and images for: Phylogenetic insights into the early spread of the SARS-CoV-2 Alpha variant across Europe
Source: Virus Evol. 2025 Jun 25;11(1):veaf030. doi: 10.1093/ve/veaf030 (PMC12223989; doi:10.1093/ve/veaf030)

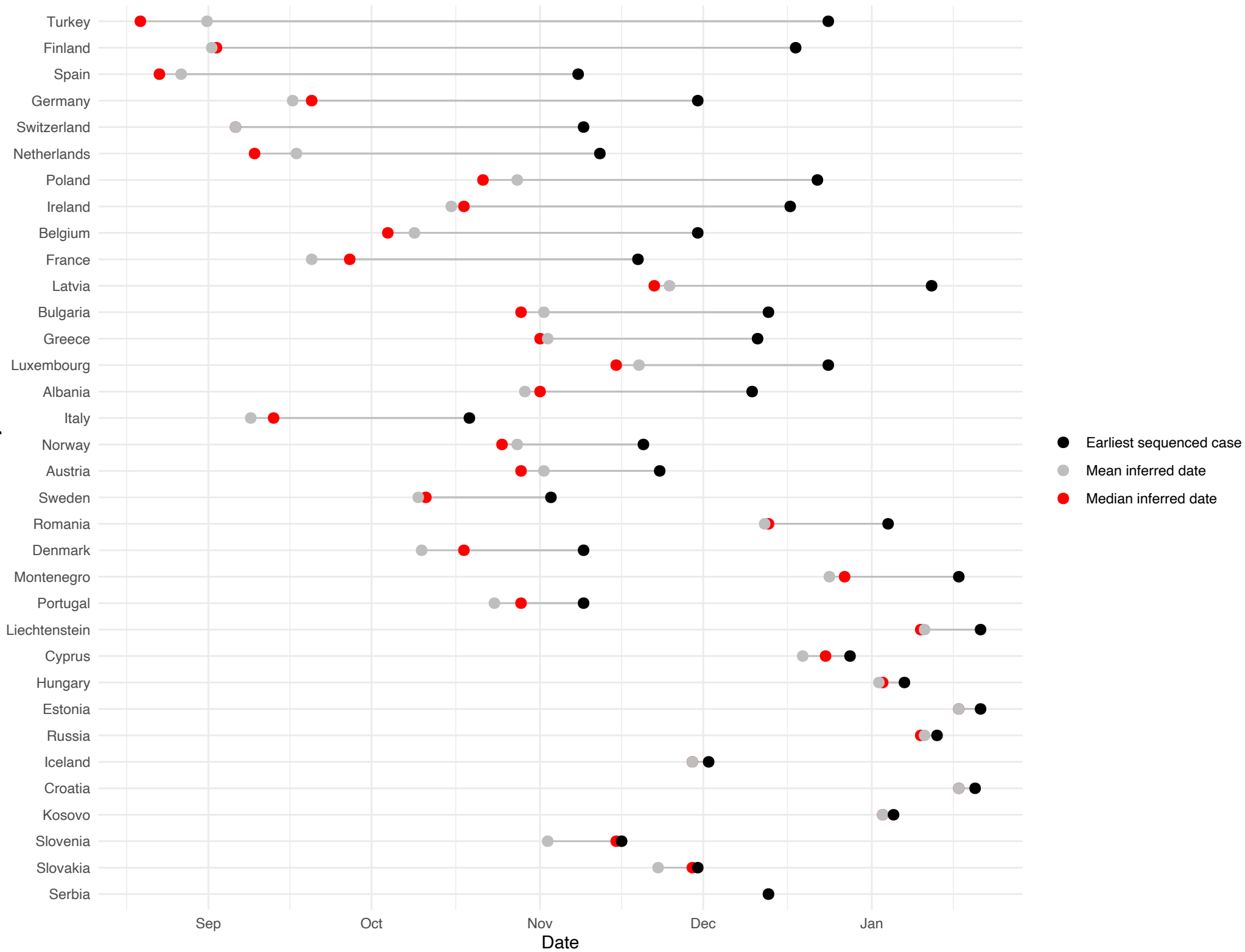

Supplement: veaf030_Supp [file veaf030_supp.zip › suppl_data/SFigure1.pdf]

A

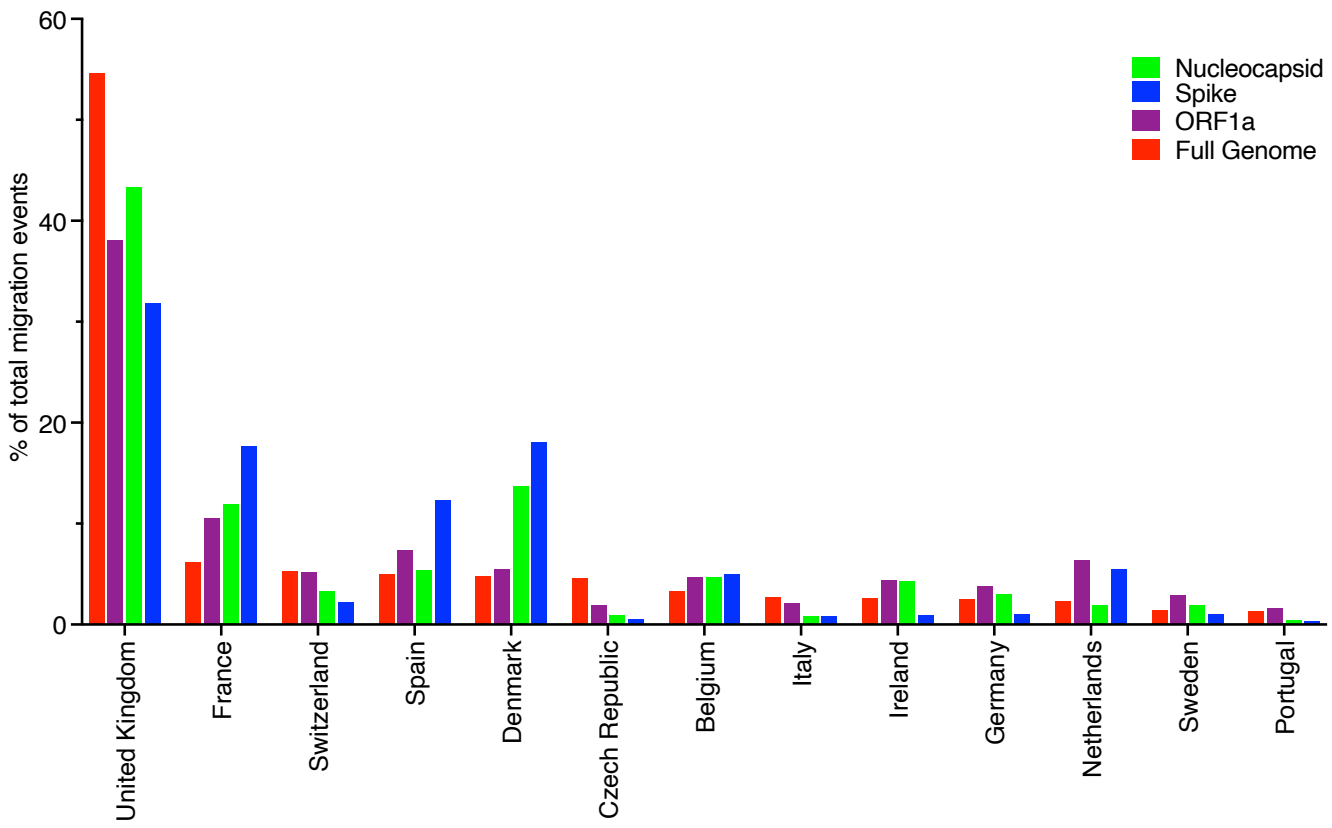

B

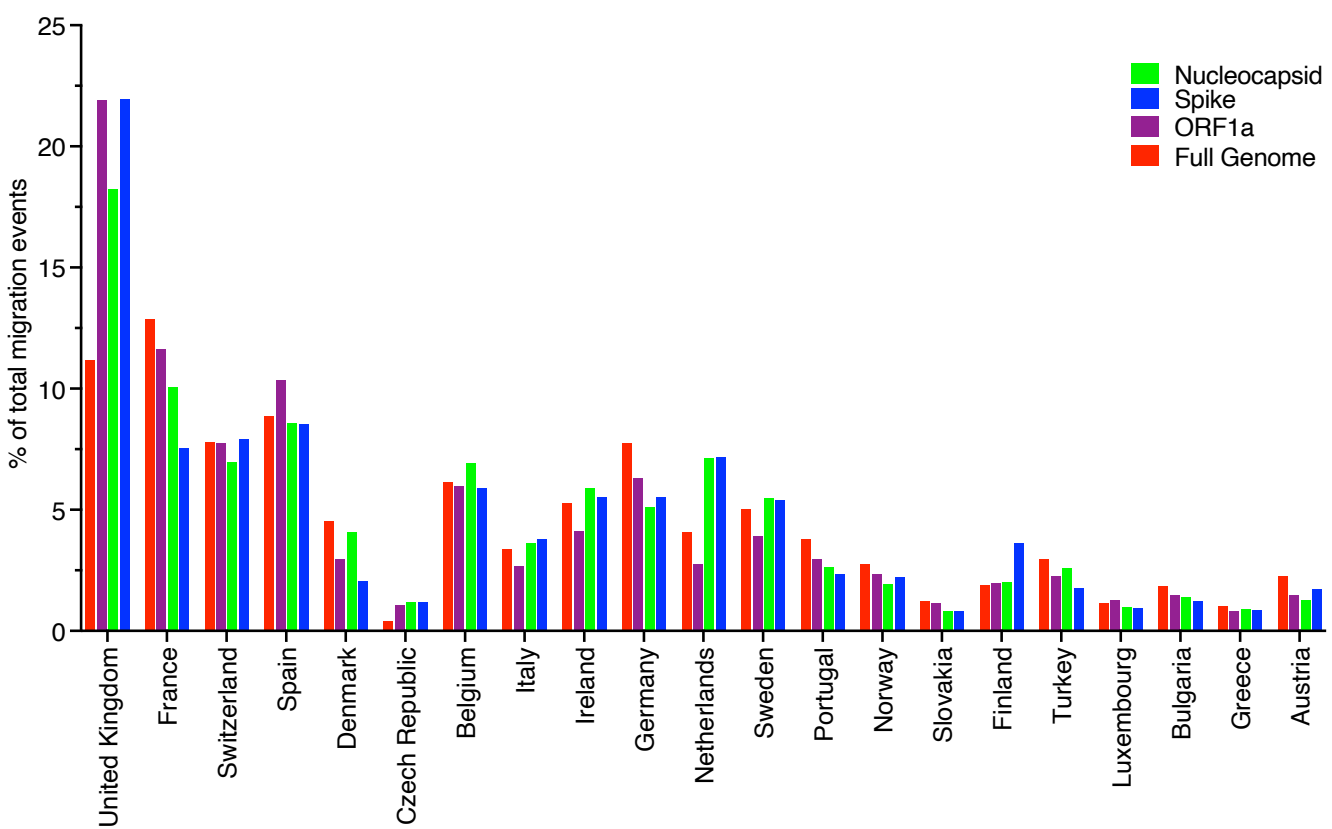

Supplement: veaf030_Supp [file veaf030_supp.zip › suppl_data/SFigure2.pdf]
